# The additional facet of immunoscore: immunoprofiling as a possible predictive tool for cancer treatment

**DOI:** 10.1186/1479-5876-11-54

**Published:** 2013-03-03

**Authors:** Paolo A Ascierto, Mariaelena Capone, Walter J Urba, Carlo B Bifulco, Gerardo Botti, Alessandro Lugli, Francesco M Marincola, Gennaro Ciliberto, Jérôme Galon, Bernard A Fox

**Affiliations:** 1 Melanoma, Cancer Immunotherapy and Innovative Therapy Unit, Istituto Nazionale Tumori Fondazione “G. Pascale”, Napoli, Italy; 2 Earle A. Chiles Research Institute, Robert W. Franz Cancer Research Center, Providence Portland Medical Center, 4805 NE Glisan, St. Portland, OR, USA; 3 Clinical Pathology Division and Translational Research Unit (TRU), Institute of Pathology, University of Bern, Bern, Switzerland; 4 Sidra Medical and Research Centre, Doha, Qatar; 5 INSERM, U872, Laboratory of Integrative Cancer Immunology, Paris, F-75006, France; 6 Université Paris Descartes, Paris, France; 7 Cordeliers Research Centre, Université Pierre et Marie Curie, Paris 6, Paris, France; 8 Department of Molecular Microbiology and Immunology, and Knight Cancer Institute, Oregon Health and Science University, Portland, OR, 97239, USA

**Keywords:** Tumor microenvironment, Tumor-infiltrating lymphocytes, Immunoprofiling, Immunotherapy, Ipilimumab

## Abstract

Recent investigations of the tumor microenvironment have shown that many tumors are infiltrated by inflammatory and lymphocytic cells. Increasing evidence suggests that the number, type and location of these tumor-infiltrating lymphocytes in primary tumors has prognostic value, and this has led to the development of an ‘immunoscore. As well as providing useful prognostic information, the immunoscore concept also has the potential to help predict response to treatment, thereby improving decision- making with regard to choice of therapy. This predictive aspect of the tumor microenvironment forms the basis for the concept of immunoprofiling, which can be described as ‘using an individual’s immune system signature (or profile) to predict that patient’s response to therapy’ The immunoprofile of an individual can be genetically determined or tumor-induced (and therefore dynamic). Ipilimumab is the first in a series of immunomodulating antibodies and has been shown to be associated with improved overall survival in patients with advanced melanoma. Other immunotherapies in development include anti-programmed death 1 protein (nivolumab), anti-PD-ligand 1, anti-CD137 (urelumab), and anti-OX40. Biomarkers that can be used as predictive factors for these treatments have not yet been clinically validated. However, there is already evidence that the tumor microenvironment can have a predictive role, with clinical activity of ipilimumab related to high baseline expression of the immune-related genes FoxP3 and indoleamine 2,3-dioxygenase and an increase in tumor-infiltrating lymphocytes. These biomarkers could represent the first potential proposal for an immunoprofiling panel in patients for whom anti-CTLA-4 therapy is being considered, although prospective data are required. In conclusion, the evaluation of systemic and local immunological biomarkers could offer useful prognostic information and facilitate clinical decision making. The challenge will be to identify the individual immunoprofile of each patient and the consequent choice of optimal therapy or combination of therapies to be used.

## 

Recent investigations of the tumour microenvironment (TME) have shown that many tumors are heavily infiltrated by a complex repertoire of inflammatory and lymphoid cells. Immune cells appear as dense infiltrates in the center of the tumoral zone, at the invasive margin of the tumor, and as lymphoid islets adjacent to the tumor. Increasing evidence supports the hypothesis that the number, type and location of tumor-infiltrating lymphocytes (TILs) in primary tumors have prognostic value, and this has led to the development of the new concept of “immunoscore”, e.g. a quantifiable measure of the infiltrate that can potentially be used as a prognostic factor [1]. This immunoscore is primarily based on the density of two lymphocyte populations, cytotoxic (CD8) and memory (CD45RO) T cells (CD3/CD45RO, CD3/CD8 or CD8/CD45RO), both in the center and the invasive margins of tumors [2-4].

**Table 1:** Differences between immunoscore and immunoprofiling

	**Immunoscore**	**Immunoprofiling**
	**Prognostic/Predictive(?)**	**Prognostic/Predictive(?)**
**Number of immune markers**	2-4	1 – Several
**Immunoscore markers**	CD3/CD8	
**Immunoscore-like markers**	CD3/CD8/CD20/FoxP3	Immune gene signatures
	CD3/CD8/CD45RO	Multiplex assays
	CD4/CD8/CD68	CD137, Galectin1, LAG-3, OX40, PD-
	CD3/CD8/CD20, CD3/GZMB	
	CD8/FoxP3	
	CD8/IL17	
	(others)	
**Possible application**	• Staging in colorectal cancer (already tested)	• Prognostic assay
	• Staging in Melanoma, Breast cancer, Ovarian cancer, NSCLC, Prostate cancer, Pancreatic cancer, Head & Neck cancer (to be defined).	• Predictive assay
		• Personalized immune-treatment

Although colorectal cancer has been the model for proof of principle during research development of the immunoscore, the relevance of the CD8 + (CD45RO or CD3) phenotype has also been shown in other tumor types, with high densities of cytotoxic and memory T cells associated with longer disease-free (after surgical resection of the primary tumor) and/or overall survival in several cancer types [5]. However, the nature of TILs is heterogeneous between tumors and so, in order to further validate the concept of the immunoscore as a prognostic factor, this needs to be characterized in other tumor types such as melanoma, renal cell, prostate, ovarian and breast cancer. Studies have already begun to investigate the relationship between Immunoscore-like markers and prognosis in cancers other than colorectal. For example, a retrospective study involving 102 women with a histologically confirmed diagnosis of early invasive breast cancer recently reported that an increased CD68 count and CD68/(CD3 + CD20) ratio at the invasive front of the carcinoma was significantly associated with occurrence of distant metastasis [6]. Further, the reverse phenotype (CD68^low^/CD4^low^/CD8^high^) was identified as an independent prognostic indicator of breast cancer survival (p < 0.001) in a retrospective study of 677 patients [7].

As well as providing useful prognostic information, the immunoscore concept also has the potential to help predict response to treatment, thereby helping improve therapeutic decisions. This predictive aspect of the quantity, quality, and distribution of the immunologic TME forms the basis for the concept of immunoprofiling, which can be defined as “using an individual’s immune system signature to predict the response to therapy” (see Table 1). The immunoprofile of an individual can be genetically determined or tumor-induced (and therefore dynamic). For example, it has previously been reported that some regional lymph nodes close to primary melanomas and breast cancers are immune-suppressed and that the degree of immune suppression is directly correlated with the closeness of the node to the tumor [8]. It has also been demonstrated that interdigitating dendritic cells are reduced and lack the complex dendrites that characterize active antigen presentation in nodes proximal to the tumor or partly replaced by tumor (e.g. sentinel lymph nodes). This could suggest nodal immune suppression due to tumor influence, mediated in part by melanoma-derived materials [9].

With the advent of immunotherapies, the predictive role of immunoprofiling will become a fundamental tool for patients’ management. Ipilimumab, a monoclonal antibody which antagonizes cytotoxic T lymphocyte-associated antigen 4 (CTLA-4), is the first in a series of immunomodulating antibodies to become available. Tumors typically develop multiple mechanisms to evade the endogenous immune response, including ‘immune checkpoints’ that can terminate immune responses after antigen activation. Immune checkpoint inhibitors, such as ipilimumab, have thus been a key target in the development of immunotherapeutic approaches for cancer. Treatment with ipilimumab has been shown to be associated with improved overall survival in patients with advanced melanoma [10]. Other immunotherapies currently being evaluated in clinical trials include anti-programmed death 1 (PD1) protein (nivolumab), anti-PD-ligand 1 (PD-L1) and anti-CD137 (urelumab), and anti-OX40 [10-13].

Biomarkers that can be used as predictive factors for ipilimumab treatment have not yet been identified. However, there is already evidence that characteristic TMEs can have a predictive role. A retrospective study in patients treated with ipilimumab suggested that clinical activity was related to high expression of the immune-related genes FoxP3 and indoleamine 2,3-dioxygenase (IDO) at baseline and an increase from baseline in TILs (at week 4) in tumor biopsies [14]. These biomarkers could represent the first potential proposal for an immunoprofiling panel in patients for whom anti-CTLA-4 therapy is being considered. However, these findings need to be confirmed in a large, prospective clinical trial. Similarly, in a recent study of the new anti-PD-1 agent nivolumab, preliminary findings suggested that the immunosuppressive PD-1 ligand, PD-L1 (B7-H1), could be a possible predictive biomarker of therapeutic response. In a subset of patients (n = 42) with various cancers, 36% with positive PD-L1 expression on the surface of tumor cells in pre-treatment tumor specimens had an objective response to treatment with anti-PD-1, while none of the patients with PD-L1-negative tumors had an objective response [15]. Again, prospective studies are needed to define the potential role of this biomarker. Other cells such as myeloid derived suppressor cells (MDSC) can be detected as infiltrating components of primary or metastatic lesions, suggesting a potential involvement in melanoma progression. Moreover, this kind of cells could have a role in predicting the response to ipilimumab [16].

This approach could form the basis for the evaluation of other immunomodulating antibody targets as possible predictive markers. Anti-PD-L1, anti-Lag3, anti-KIR, anti-TIM-3, anti-GITR, anti-OX40 and anti-CD137 represent the future of immunotherapy and it may be that assessment of the relevant markers can help define the individual immune system profile. This can then be used to help guide treatment choices with the different immunotherapies, used either alone or in combination.

Effective Immunoprofiling will not only consider the surface receptors of immune system cells, but also the presence of ectopic immune structures such as the tumor-localized ectopic lymph node-like structures (TL-ELNs) [17]. Recently, it was demonstrated that a 12-chemokine gene expression signature (CCL2, CCL3, CCL4, CCL5, CCL8, CCL18, CCL19, CCL21, CXCL9, CXCL10, CXCL11, and CXCL13) is strongly associated with the presence of TL-ELNs, and with a better patient outcome in colorectal cancer and melanoma [18].

In conclusion, increasing evidence supports the view that cancer development is strongly influenced by the host immune system. The evaluation of systemic and local immunological biomarkers could offer useful prognostic information and facilitate clinical decision making about the need for specific therapies, and the ‘immunoscore’ concept is quickly gaining momentum with additional trials, research activity, and retrospective validations. More patient-specific immunoprofiling represents yet another step toward personalized medicine, incorporating tests that inform clinicians and patients toward clear decision-making.

## Competing interest

PAA is consultant for Merck Sharp & Dohme and Bristol-Myers Squibb. He has participated in advisory boards for Bristol-Myers Squibb, Merck Sharp & Dohme, Roche-Genentech, GlaxoSmithKline, Amgen, Celgene, Medimmune, and Novartis and has received honoraria from Bristol-Myers Squibb, Merck Sharp & Dohme, and Roche-Genentech. He has received research support for immunoscore projects from Bristol-Myers Squibb and Ventana-Roche. WU has received honoraria from Bristol-Myers Squibb. JG is a consultant for Roche. BAF has received research support for immunoscore projects from Bristol-Myers Squibb and Ventana-Roche. All other authors have no competing interest.

## Authors’ contributions

All Authors drafted and approved the final manuscript.
